# Imprinting Disorders and Epigenetic Alterations in Children Conceived by Assisted Reproductive Technologies: Mechanisms, Clinical Outcomes, and Prenatal Diagnosis

**DOI:** 10.3390/genes16101242

**Published:** 2025-10-21

**Authors:** Antonella Gambadauro, Valeria Chirico, Francesca Galletta, Ferdinando Gulino, Roberto Chimenz, Giorgia Serraino, Immacolata Rulli, Alessandro Manganaro, Eloisa Gitto, Lucia Marseglia

**Affiliations:** 1 Neonatal and Pediatric Intensive Care Unit, Department of Human Pathology in Adult and Developmental Age “Gaetano Barresi”, University Hospital “G. Martino”, 98124 Messina, Italy; gambadauroa92@gmail.com (A.G.); francygall.92@gmail.com (F.G.); immacolata.rulli@polime.it (I.R.); alessandro.manganaro@polime.it (A.M.); eloisa.gitto@unime.it (E.G.); lmarseglia@unime.it (L.M.); 2 Pediatric Nephrology and Dialysis Unit, Department of Human Pathology in Adult and Developmental Age “Gaetano Barresi”, University Hospital “G. Martino”, 98124 Messina, Italy; valeriachirico@hotmail.it (V.C.); roberto.chimenz@unime.it (R.C.); 3 Unit of Gynecology and Obstetrics, Department of Human Pathology in Adult and Developmental Age “Gaetano Barresi”, University Hospital “G. Martino”, 98124 Messina, Italy; 4 University of Messina, 98122 Messina, Italy; giorgiaserraino00@gmail.com

**Keywords:** Angelman syndrome, ART, assisted reproductive technology, Beckwith–Wiedemann syndrome, epimutation, imprinting disorder, ICSI, IVF, Prader–Willi syndrome, Silver–Russell syndrome

## Abstract

Assisted reproductive technologies (ARTs) have revolutionized infertility treatment, leading to the birth of over 10 million children worldwide. Despite their success, increasing concerns have been expressed regarding the potential long-term outcomes of ART-conceived individuals, particularly in relation to imprinting disorders (IDs). IDs result from the abnormal expression of imprinted genes, which are expressed in a parent-of-origin-specific manner and regulated by epigenetic mechanisms (e.g., DNA methylation). Disruption of these processes, through environmental, genetic, or procedural factors, can lead to disorders such as Beckwith–Wiedemann syndrome (BWS), Silver–Russell syndrome (SRS), Angelman syndrome (AS), and Prader–Willi syndrome (PWS). These syndromes are characterized by distinct clinical features, including growth abnormalities, neurodevelopmental delay, endocrine dysfunction, and cancer predisposition. ART procedures, especially ovarian hyperstimulation, in vitro fertilization (IVF), and embryo culture, coincide with critical periods of epigenetic reprogramming and may contribute to epimutations in imprinting control regions. In this review, we explored epidemiology, molecular mechanisms, and prenatal diagnostic strategies related to these four IDs in the context of ART. The findings suggest a higher prevalence of BWS and SRS in ART-conceived children. The data regarding AS and PWS are more controversial, with conflicting results across populations and methodologies. Although a causal link between ART and IDs remains debated, evidence suggests the potential contribution of ART procedures to epigenetic dysregulation in susceptible individuals. Further large-scale, methodologically rigorous studies will be essential to clarify this association and inform safer ART practices.

## 1. Introduction

Assisted reproductive technologies (ARTs) are in vitro procedures developed to provide a helpful alternative for infertile couples to have children [1]. Globally, infertility affects millions of people and the number of children born from ARTs exceeds 10 million [2,3]. ARTs involve more procedural steps: ovarian stimulation; collection of gametes from ovaries; in vitro fertilization (IVF) with sperm; embryo culture; and embryo transfer (ET) to the female reproductive tract. These procedures can involve egg or sperm donors and/or gestational carriers [4,5]. Over time, ARTs have been improved and have become more successful in addressing diverse infertility-related problems. However, ARTs can still create concern in families, especially considering that knowledge of the long-term outcomes for babies conceived using these procedures is still limited [6].

Imprinted genes are expressed in a monoallelic and parental origin-specific manner. This means that either the maternal or paternal allele is expressed, while the other copy is silenced [7]. Imprinted genes play crucial roles in prenatal and postnatal growth, neuromotor development, endocrine and metabolic regulation, and puberty onset [8]. Genomic imprinting is pivotal for the regular and normal development of the embryo and the fetus [7]. Imprinting disorders (IDs) are the result of the aberrant expression of imprinted genes, and they comprise a group of diseases caused by an epigenetic, genetic, or cytogenetic abnormality of a specific locus/loci [9]. Previously, a possible correlation between ARTs and rare IDs, such as Beckwith–Wiedemann syndrome (BWS), Russell–Silver syndrome (SRS), Angelman syndrome (AS), and Prader–Willi syndrome (PWS), was suggested [10,11,12,13,14,15]. However, the relationship between ARTs and IDs is still debated. Many studies have reported that specific procedures (such as ovarian stimulation and IVF) may alter correct genomic imprinting, leading to an increased risk of IDs [16,17,18]. Furthermore, the role of environmental risk factors in determining this association is unclear.

In this study, we reviewed the existing literature on the association between ARTs and IDs. In particular, we analyzed the epidemiology, clinical signatures, and prenatal diagnostic workup of the four IDs mainly associated with ARTs in previous studies (BWS, SRS, AS, and PWS). By combining the key terms “assisted reproductive technologies” OR “ART” AND “gene disorders” OR “imprinting disorders” OR “Beckwith-Wiedemann syndrome” OR “Russell-Silver syndrome” OR “Angelman syndrome” OR “Prader-Willi syndrome” in a computerized search of PubMed limited to the last 10 years, we provided an overview based on a critical evaluation without standardized methodologies or statistical analyses.

## 2. Mechanisms of Imprinting Disorders

To date, roughly 150 imprinted genes have been described in humans [9]. Imprinted genes are mainly expressed in clusters (imprinted regions), which are regulated by imprinting control regions (ICRs) [19]. The parental origin-specific expression of imprinted genes is determined by differing ICR methylation. DNA methylation is an important epigenetic modification that occurs principally in cytosines within CpG dinucleotides [20]. ICR methylation is transmitted via gametes during fertilization, and it is kept stable in the somatic cells during embryogenesis [19]. The allele from one parent is unmethylated at the ICR level and transcriptionally activated, while the allele from the other parent is methylated at the ICR level and transcriptionally silenced [20].

**Figure 1:**
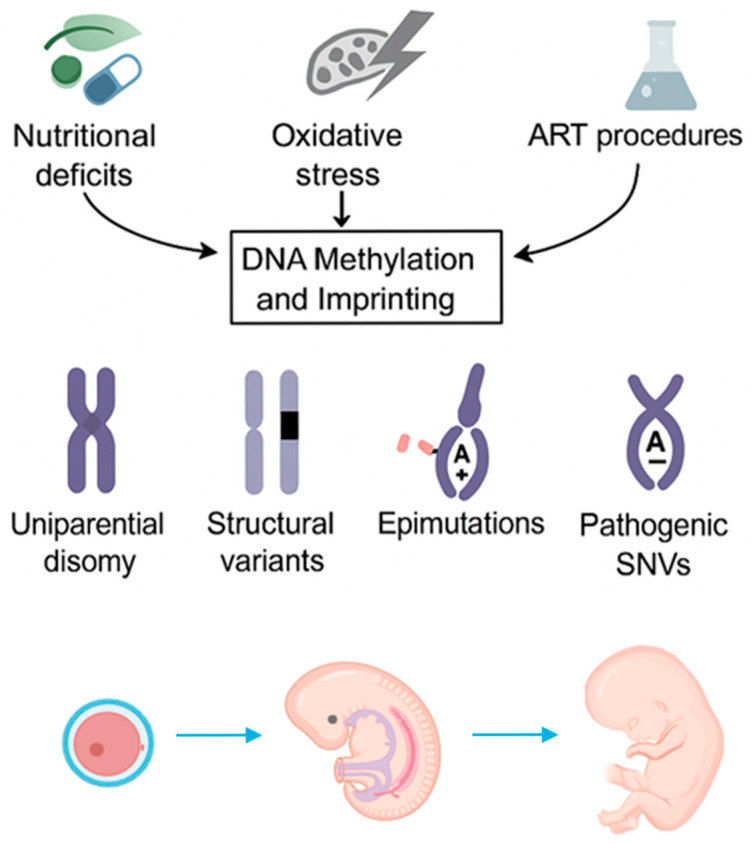
Mechanisms and environmental influences in the pathogenesis of imprinting disorders.

The aberrant expression of imprinted genes, due to some error in the removal of the parental imprint, the establishment of the parental origin-specific methylation imprint, or the maintenance of the methylation imprint during fertilization and embryogenesis, can lead to IDs [8]. Currently, four genetic alterations have been associated with IDs: 1. uniparental disomy (UPD) of chromosomes, describing the abnormal inheritance of a chromosomal region (including imprinted genes) from the same parent; 2. pathogenic structural variants involving the imprinted region (e.g., deletions or duplications); 3. pathogenic single-nucleotide variants (SNVs) of imprinted genes; and 4. epimutation, showing an aberrant ICR methylation signature, which leads to abnormal expression of imprinted genes (Figure 1) [18,20].

Both genetic and environmental factors can influence the imprinting process [9]. Among environmental factors, nutritional status, oxidative stress, and endocrine disruptors may impact genomic imprinting, increasing the risk of IDs (Figure 1) [9]. For example, a lack of free methyl donors, such as S-adenosylmethionine (SAM), may impact DNA methylation. In particular, deficient methyl-intake diets, low folate levels, and genetic variants of proteins involved in one-carbon (1C) metabolism can affect the methylation pattern of a specific imprinted region (11p15.5) [21,22,23]. Recent studies suggest the presence of cellular energy status sensors, which protect cells against oxidative stress by regulating epigenetic mechanisms. Sirt-1 (sirtuin-1) is a nicotinamide adenine dinucleotide (NAD)-dependent deacetylase (HDAC) that regulates the cellular stress response [24]. In a murine embryonic stem cell (ESC) model, Sirt-1 was shown to prevent abnormal DNA methylation at imprinted loci by antagonizing Dnmt3l, a positive regulator of methylation [24]. By considering endocrine disruptors, prenatal exposure to bisphenol A (BPA) was shown to be related to imprinting and methylation anomalies in both murine placenta and developing gametes [25,26,27,28]. Advanced maternal age was also described as an independent risk factor, particularly promoting the development of aneuploid UPD independently of ART procedures. Increased chromosomal segregation errors in aging oocytes can contribute to trisomy rescue events, leading to UPD and, consequently, IDs. [29]. Eight syndromes have epimutation as a genetic cause, which can be exasperated by environmental factors [8,20].

In addition to environmental exposures, each step of ART may affect imprint establishment and maintenance at different developmental stages. ARTs are usually attributed to male or female infertility and comprise several procedures, e.g., ovarian hyperstimulation, IVF, intracytoplasmic sperm injection (ICSI), embryo culture, and ET. All these techniques coincide with crucial events in epigenome reprogramming and can be associated with epimutation in ICRs, leading to IDs [10,12,30,31]. The risk of IDs is not related to a single intervention but to the cumulative impact of multiple ART procedures applied during highly vulnerable developmental stages. This cumulative risk emphasizes the need to optimize each procedural step to minimize epigenetic disruption.

### 2.1. Ovarian Hyperstimulation

Ovarian hyperstimulation involves high-dose hormonal treatments and is used to obtain multiple oocytes, occurring during gametogenesis. Experimental studies in mice have shown that this procedure is linked to altered oocyte gene expression, reduced embryo maturation, and delayed embryonic development [32,33]. In humans, transcriptomic studies indicate that high-dose gonadotropin exposure may interfere with DNA methylation reprogramming and imprint acquisition. A recent systematic review further empathized that ovarian stimulation protocols, especially when involving high gonadotropin doses, may contribute to aberrant methylation patterns and increase susceptibility to IDs [34]. Evidence also suggests that ovarian hyperstimulation protocols can induce oxidative stress and alter one-carbon metabolism pathways, altering DNA methylation [35,36]. These protocols may increase epimutations at imprinted loci (e.g., PEG1, KCNQ1, and ZAC) compared to oocytes obtained from a natural ovulatory cycle [37,38].

### 2.2. In Vitro Fertilization (IVF) and Intracytoplasmic Sperm Injection (ICSI)

Both IVF and ICSI involve mechanical and chemical manipulation of gametes and zygotes. This manipulation occurs during a sensitive phase of epigenetic reprogramming, increasing the risk of aberrant methylation [39]. IVF/ICSI procedures have an increased risk of adverse perinatal outcomes, including abnormal placentation or low birth weight [40,41]. ICSI, which bypasses natural sperm selection, has been most consistently linked to multi-locus imprinting disturbances (MLIDs), especially in disorders such as BWS and SRS [42]. Recent reviews emphasize that ICSI is more frequently associated with IDs than conventional IVF and raise concerns about its overuse beyond strict male-factor infertility [38,41]. ICSI-conceived infants have an approximately 1% of risk of chromosomal alteration or aneuploidy, while naturally conceived children and kids conceived with standard IVF insemination have a lower risk (0.2% and 0.7%, respectively) [41,43].

### 2.3. Embryo Culture

In vitro culture exposes embryos to non-physiological conditions (e.g., temperature, pH, oxygen, and culture media composition). Culture conditions and culture medium composition are the two main factors affecting embryo development during in vitro culture [39]. Non-physiological oxygen concentrations (20% or 5%), exposure to suboptimal pH, and variations in amino acid or methyl donor content in culture media can alter methylation at ICRs and affect blastocyst morphology and gene expression [44,45,46]. Both animal and human studies have shown that culture media may promote changes in cellular, developmental, and metabolic pathways [47,48]. Furthermore, when the embryo is handled in vitro and cultured for up to six days in culture media, ARTs may alter DNA methylation, which takes place during the preimplantation stages of embryonic development, leading to IDs [49]. Some studies have compared different types of embryo culture media (global medium (LifeGlobal) versus single-step medium (Irvine Scientific) or human tubal fluid versus G5 culture medium) and have not found any statistically significant differences in DNA methylation patterns [50,51]. Recently, some concern was expressed regarding extending embryo culture to the blastocyst stage, which may increase the risk of imprinting errors considering that significant epigenetic reprogramming takes place in this stage [52].

### 2.4. Cryopreservation

Cryopreservation, particularly vitrification, subjects the embryos to osmotic and oxidative stress [8]. Despite early data suggesting a low impact on imprinting processes, recent studies have identified altered methylation signatures associated with these protocols. Cryopreservation allows for oocyte and embryo freezing and storage in liquid nitrogen, interrupting all biological activities and maintaining their viability [53]. Current vitrification protocols adopt a cryoprotectant mixture (e.g., ethylene glycol, dimethyl sulfoxide, sucrose, or trehalose), which may be toxic for the embryo [38]. In particular, ethylene glycol and dimethyl sulfoxide can alter DNA methylation and induce structural changes in chromatin and DNA double-strand breaks [54]. Cryopreserved embryos show distinct epigenetic profiles compared with fresh embryos, though clinical consequences are still under investigation [55]. The choice of cryopreservation technique strongly influences the epigenetic outcome, with vitrification generally considered more disruptive than slow freezing [38].

### 2.5. Embryo Transfer (ET)

ET coincides with implantation and early placentation, processes that are crucial for epigenetic stability [39]. Suboptimal endometrial conditions, repeated transfers, or mechanical stress may interfere with methylation maintenance. Evidence remains limited, but reviews suggest that endometrial receptivity and the maternal hormonal environment can modulate epigenetic stability after ET [55].

### 2.6. Combined ART Procedures

Exposure to multiple and sequential ART procedures may amplify risks for IDs and developmental abnormalities. Experimental and clinical data suggest that combined interventions can create additive or synergistic stress for gametes and embryos compared to isolated procedures. However, causality is difficult to establish due to confounding factors such as parental age and infertility etiology [29,56]. When ovarian hyperstimulation is followed by ICSI, the mechanical injection bypasses natural sperm selection and exposes oocytes to additional oxidative and mechanical stress. Subsequent in vitro embryo culture may further affect genomic imprinting during critical windows of epigenetic reprogramming. Cryopreservation, despite technical advances in vitrification, can also induce osmotic and oxidative stress, with potential damage to imprinting stability and placental development [46]. A study conducted on a mouse model to assess placental morphology and methylation profiles revealed that ET and superovulation procedures both induce placental morphological alterations, while IVF was specifically associated with the hypomethylation of ICRs and a global reduction in DNA methylation levels [57]. Furthermore, embryo culture and vitrification parameters (e.g., temperature, osmolality, and cryoprotectant concentration) interact dynamically with earlier ovarian and fertilization environments, potentially changing methylation patterns and key imprinted loci [56]. Considering these complex interactions, ART protocols should be optimized to minimize cumulative stress across procedures. Personalized ART planning, considering patient-specific infertility causes and limiting unnecessary procedures, may mitigate epigenetic risk. Further studies are needed to assess the combined impact of ART procedures and to help with pre-ART counseling.

Environmental factors (e.g., nutritional status and oxidative stress) can influence the imprinting process. ART procedures are also associated with the alteration of DNA methylation. Four genetic alterations have been associated with imprinting disorders: 1. uniparental disomy (UPD) of chromosomes, describing the abnormal inheritance of a chromosomal region from the same parent; 2. pathogenic structural variants involving the imprinted region; 3. pathogenic single-nucleotide variants (SNVs) of imprinted genes; and 4. epimutation, showing an aberrant ICR methylation signature, which leads to abnormal expression of imprinted genes.

## 3. ART-Related Imprinting Disorders

### 3.1. Beckwith–Wiedemann Syndrome

BWS is a congenital overgrowth disorder with a prevalence of 1:10,340 in naturally conceived babies [58]. This condition is characterized by variable associations between neonatal macrosomia, postnatal overgrowth, hyperinsulinemic hypoglycemia, abdominal wall defects (e.g., omphalocele), macroglossia, organomegaly, auricular deformities, nephro-urological anomalies, and cancer predisposition (e.g., Wilms tumor) [59,60,61]. Over 80% of BWS patients have an epigenetic defect in the imprinted chromosomal region 11p15.5, comprising ICR2 hypomethylation (50% of cases), chromosome 11 paternal UPD (20% of cases), and the gain-of-methylation of ICR1 [62]. More rarely, BWS can be associated with genetic defects, such as a loss of function of the CDKN1C variant or rearrangements in the 11p15.5 region [63].

The overall percentage of children with BWS conceived with ARTs is above that observed in the general population, ranging from 4% to 15% [10,11,12,64]. However, some studies have reported no association between ART and BWS [65]. A wide register-based cohort study conducted in children born in Denmark and Finland between 1990 and 2014 revealed a 2.8 higher risk of developing BWS in ART children compared to the naturally conceived group [49]. These authors reported that mothers of children with IDs were slightly older than mothers of children without IDs (mean maternal age: 35.8 ± 4.2 years versus 33.7 ± 4.3 years for the ART group and 31.1 ± 5.4 years versus 30.0 ± 5.1 years for the natural conception group) [49]. The importance of maternal age was described in another study by Hara-Isono et al.: the authors demonstrated that ARTs performed at over 30 years of age can facilitate the occurrence of IDs (particularly BWS and SRS) due to the increased risk of epimutations [66]. An Italian study comparing children born after ART and those conceived naturally reported a 10-fold increased risk of BWS in the first group [67]. However, in some studies the percentage risk of developing BWS seems to be related to the specific type of ART, particularly the combined use of intracytoplasmic sperm injection and cryopreserved embryos [6]. By considering molecular bases, BWS patients conceived through ARTs seem to develop ICR2 hypomethylation more frequently (>85% of cases) compared to naturally conceived BWS children [68,69]. Other epigenetic defects exhibit lower expression in BWS children conceived via ARTs compared to those naturally conceived: for example, the risk of chromosome 11 paternal UPD in children with BWS conceived via ARTs ranges from 3.4% to 18.5% [70].

### 3.2. Angelman Syndrome

AS is a rare neurogenetic disorder affecting nearly 1:15,000 people [71]. This rare disease is characterized by severe intellectual disability, developmental delay, speech impairment, and ataxia [72]. AS is mainly caused by loss of function of the UBE3A gene, located on chromosome 15q11.2-q13. UBE3A is normally inherited from each parental allele, but only the maternal copy of the gene is expressed in the cells [73]. The loss of function of the UBE3A gene in AS is generally a consequence of microdeletions on the maternal chromosome (65–75% of cases) [74]. However, SNVs, UPDs, or epimutation (<5% of cases) have also been described [75].

Subfertile couples treated with ICSI or ovarian hyperstimulation show a doubled risk of having a child with AS when compared with subfertile couples who do not have any treatment [30,76]. In babies born from ICSI, hypomethylation of the SNRPN locus on the maternal chromosome was described [30]. In individuals without AS, the SNRPN allele is normally methylated, and the recognition of this anomaly in patients with AS born from ICSI revealed a sporadic imprinting defect [30]. However, recent studies have negated the association between AS and ARTs, showing no significant increase in prevalence compared to naturally conceived infants [13,42,49]. Further studies are needed to evaluate the actual association.

### 3.3. Prader–Willi Syndrome

PWS is a neurobehavioral ID that affects 1:15,000–1:30,000 individuals [77]. It is characterized by decreased fetal movement and neonatal hypotonia, feeding difficulties, failure to thrive, mild intellectual disability, neurobehavioral anomalies (e.g., obsessive–compulsive behaviors and attention-deficit and hyperactivity symptoms), characteristic facial features (almond-shaped palpebral fissures, bitemporal narrowing, and strabismus), and hypogonadism (genital hypoplasia and delayed or incomplete pubertal development) [78,79]. PWS is caused by the complete absence of expression of the paternally imprinted genes in the 15q11.2-q13 region. The genetic mechanisms leading to PWS are paternal deletion of this region (65–75% of cases), maternal UPD (20–30% of cases), and an ICR defect (2–5% of cases) [77,78]. The affected imprinting genes are MKRN3, MAGEL2, NECDIN, and SNURF-SNRPN, as well as six small nucleolar RNAs (snRNAs) [80]. In PWS, paternal copies of the genes are typically expressed, while the maternal copies of these genes are silenced due to parent-of-origin-specific imprinting [78].

The recent literature provided contrasting results about the relationship between ARTs and PWS. A study on Danish and Finnish cohorts did not reveal any significant increase in the frequency of PWS among ART populations [49]. However, a study on a Japanese cohort revealed a frequency of PWS 3.4-fold higher in babies conceived with ARTs compared to those naturally conceived [42]. Furthermore, a maternal age ≤37 years was significantly associated with an increased rate of DNA methylation errors in PWS infants conceived with ARTs compared to those naturally conceived [42]. The increased rate of methylation errors, together with the higher rate of maternal UPD, among PWS patients born from ARTs was also reported in a study on a wide American cohort [81]. The different rates of PWS infants between several populations conceived with ARTs may be the result of ethnic differences [42,49,81]. However, further studies are needed to determine the role of ethnic factors.

### 3.4. Silver–Russell Syndrome

SRS is a rare genetic disorder with an incidence ranging from 1:30,000 to 1:100,000 in the general population [82]. It is characterized by low birth weight, growth delay in the postnatal period, characteristic facial features (triangular face structure, prominent head, and small jaw), and body asymmetry [83]. It is a complex disorder, and the most common causative mechanism is a methylation defect (mainly hypomethylation of the H19/IGF2 ICR) in the 11p15.5 region (30–60% of cases) [82]. However, maternal UPD of chromosome 7 is also reported in SRS patients (5–10% of cases) [84].

Estimates of SRS births related to ARTs are inhomogeneous in the literature. Two nationwide epidemiological studies conducted in Japan (2009 and 2015) reported 10-fold and 8.9-fold increased frequencies of SRS associated with ART, respectively [15,42]. Furthermore, all SRS patients conceived via ARTs reported DNA methylation errors, while SRS patients naturally conceived showed both DNA methylation errors and UPD [15,42]. A study on murine models reported that enhanced methylation rates are the result of ART, resulting in low birth weight due to the use of improper sperm samples and IVF environmental factors (e.g., culture medium components) [85]. Both IVF and ICSI, occurring during critical phases of epigenetic reprogramming, may impair the establishment or maintenance of correct DNA methylation [39,86].

### 3.5. Other Imprinting Disorders

Other IDs have been described in the literature in association with ARTs. However, studies about the effective incidence and distribution of these disorders in patients born from ARTs are still rare.

Temple Syndrome (TS14) is caused by imprinting defects at 14q32 involving the MEG3/DLK1:IG-differentially methylated region (DMR). It is mainly related to maternal UPD of chromosome 14 or loss-of-methylation (LOM) of the MEG3/DLK1:IG-DMR [8,19]. Data on the association between TS14 and ARTs are limited. However, its etiopathogenetic mechanism is consistent with epimutations seen in ART-conceived individuals. TS14 is characterized by fetal and neonatal growth retardation, neonatal hypotonia, feeding difficulties in infancy, truncal obesity, and precocious puberty [8,19].

Transient Neonatal Diabetes Mellitus (TNDM) is associated with imprinting defects at chromosome 6q24, mainly involving the PLAGL1:alt-TSS-DMR. It is mainly related to paternal UPD, duplication of chromosome 6, or LOM of the PLAGL1:alt-TSS-DMR [8,19]. Data from a British study did not find a statistically significant increased frequency of TNDM in children born from ART, but data were too sparse to provide a statistically meaningful estimation of the ART-TNDM correlation [13,87]. TNDM is characterized by low birth weight, hyperglycemia without ketoacidosis, macroglossia, and abdominal wall defects [8,19].

Kagami–Ogata Syndrome (KOS14) is associated with imprinting defects at chromosome 14q32 involving the MEG3/DLK1:IG-DMR. It is mainly related to paternal UPD of chromosome 14 or gain-of-methylation (GOM) at MEG3/DLK1:IG-DMR or maternal deletion at chromosome 14q32 [8,19]. Data on the association between TS14 and ARTs are limited. However, considering its etiopathogenetic mechanism, it can be assumed that epimutations occurring in this syndrome could be associated with ARTs. KOS14 is characterized by respiratory failure at birth, polyhydramnios, abdominal wall defects, a small bell-shaped thorax, coat-hanger ribs, a narrow chest wall, feeding difficulties, intellectual disability, and elevated tumor risk (hepatoblastoma) in childhood [8,19].

Pseudohypoparathyroidism (PHP) involves epigenetic defects in the GNAS locus at chromosome 20, especially LOM at maternally methylated regions. The GNAS locus was identified as an epigenetically vulnerable region in ART-conceived children [8,19]. However, large epidemiological data about the association between ARTs and PHP are limited. PHP is characterized by resistance to parathyroid hormone (PTH) and Albright hereditary osteodystrophy (AHO) [8,19].

### 3.6. Multi-Locus Imprinting Disturbances (MLIDs)

A growing body of evidence indicates that ART-conceived children may present with multi-locus imprinting disturbances (MLIDs), in which epimutations affect multiple ICRs simultaneously rather than a single locus [88,89]. MLIDs have been described in patients with BWS, SRS, and other rare IDs and may contribute to the wide phenotypic variability observed in ART-associated cases [90,91]. MLIDs are related to global impairment of imprint maintenance during preimplantation development, a stage highly sensitive to embryo culture conditions and paternal factors. Notably, ICSI/IVF has been particularly associated with MLIDs, possibly due to sperm with abnormal imprinting signatures bypassing natural selection barriers [92]. Recognition of MLID is clinically relevant, as it may predispose to broader neurodevelopmental, metabolic, or tumor-related risks compared to single-locus defects [93].

## 4. Prenatal Diagnosis

### 4.1. Preimplantation Genetic Testing (PGT)

The augmented incidence of some of the IDs mentioned in our review among ART-conceived individuals highlights the importance of prenatal screening for these disorders. Preimplantation genetic testing (PGT) evaluates the DNA content of oocytes and embryos for genetically normal embryo selection. Six groups of screening procedures are described in the literature: 1. PGT-aneuploidy, which screens all 46 chromosomes regarding numerical abnormalities; 2. PGT-monogenic, which diagnoses monogenic or single-gene disorders; 3. PGT-structural rearrangement, which detects translocations or inversions inherited from one or both parents; 4. combined PGT, which allows for simultaneous diagnosis and screening of all abnormalities; 5. extended PGT, which investigates multifactorial and polygenic disorders; and 6. non-invasive PGT, which can be performed by blastocentesis (i.d., aspiration of the blastocyst fluid) or by analyzing exhausted culture media [94]. The most recent literature still does not suggest the overall extension of prenatal screening procedures to all infants conceived via ARTs but limits specific investigations in the case of ID-associated findings on fetal ultrasound [95]. However, some disorders (such as PWS and AS) are not related to specific ultrasound abnormalities.

### 4.2. Disorder-Specific Prenatal Features

The most common indication of prenatal testing for IDs is abnormal fetal growth, mainly for BWS and SRS and less frequently for TS14 and KOS14. In Table 1, we summarize the main IDs related to ARTs, reporting specific prenatal findings. In the case of fetal growth alteration, it is important to exclude the presence of IDs through appropriate molecular genetic analysis. BWS is associated with fetal overgrowth, while SRS, TS14, and KOS14 are related to intrauterine growth restriction (IUGR). Carli et al. proposed a clinical scoring system for prenatal molecule investigations in patients with features potentially associated with BWS [96]. In this scoring system, major, minor, and supportive features are represented by macroglossia, omphalocele, placental mesenchymal dysplasia, family history of inheritable BWS, and BWS-related typical tumors (major features); macrosomia, visceromegaly, polyhydramnios, and placentomegaly (minor features); and nephroureteral anomalies, elevated beta-human chorionic gonadotropin (β-hCG)/alpha-fetoprotein (α-FP)/pregnancy-associated plasma protein A (PAPP-A), monozygotic twinning, and pregnancy from ARTs (supportive features) [96]. A score ≥3 points signifies the need to perform prenatal molecular testing for BWS [96]. Azzi et al. proposed a postnatal clinical scoring system to optimize SRS diagnosis [97]. This score was based on six criteria: low birth weight and/or birth length, postnatal growth retardation, macrocephaly at birth, protruding forehead, body asymmetry, and feeding difficulties [97]. The presence of four of the six criteria suggested a clinical diagnosis of SRS, needing a molecular confirmation [97]. Antenatally, SRS can be suspected in the presence of early IUGR, possibly in combination with macrocephaly and facial dysmorphism. However, few studies have analyzed prenatal diagnosis of SRS due to the non-specific intrauterine phenotype and the low prevalence of the disorder [98,99,100]. TS14 is rarely diagnosed prenatally due to non-specific features. Characteristic findings are oligohydramnios, growth restriction, reduced fetal movements, small placenta, and preterm birth. Ioannides et al. reported that TS14 infants mainly showed IUGR and low weight at birth [101]. In a study by Li et al., prenatal features of KOS14 were mainly represented by polyhydramnios, abdominal wall defects, exomphalos, a small thorax, and short limbs [102].

Previous studies described non-specific prenatal features for the suspicion of PWS. In particular, this disorder should be excluded in the presence of reduced fetal movements and polyhydramnios. In the neonatal period, this ID is one of the most important differential diagnoses in the presence of a floppy infant with severe hypotonia [103]. AS is not characterized by specific prenatal symptoms, but this disorder should be suspected in neonates or infants with severe developmental delay [104]. Antenatally, the association between IUGR, macroglossia, and abdominal wall defects is needed to exclude TNDM. Meanwhile growth abnormalities are present in different types of PHP (macromia in PHP1B and IUGR in PHP1A).

### 4.3. Genetic Counseling

Genetic counseling is based on the discussion of the clinical phenotype of the offspring, the underlying molecular cause, the recurrence risk, and the potential clinical outcomes. Furthermore, it focuses on prenatal management options, including termination of pregnancy. The prenatal detection of IDs is often challenging due to their variable molecular basis, mosaicism, and non-specific ultrasound findings [95]. The most common IDs diagnosed in the prenatal period are SRS and BWS. Prenatal methylation analyses are useful in the presence of abnormal ultrasound findings. Families with a previous history of IDs, recurrent miscarriages (especially with evidence of hydatidiform moles), or conceptions via ARTs should receive targeted genetic assessment, including methylation studies and UPD testing of relevant loci. Counseling should also explain the possibility of MLID and maternal effect mutations, which may increase the recurrence risk [95]. A multidisciplinary approach involving reproductive specialists, geneticists, and neonatologists is crucial to ensure informed decision-making and appropriate follow-up.

## 5. Long-Term Outcomes

Literature research suggests that ART-conceived children may experience a wide range of long-term outcomes. These include differences in growth patterns, endocrine dysfunction, increased metabolic and cardiovascular risks, and neurocognitive and behavioral anomalies. Such outcomes derive from combined factors that include high hormonal exposure, gamete and embryo manipulation, and epigenetic alterations occurring during critical stages of early development.

### 5.1. Cardiometabolic Outcomes

Several studies indicate that children and adolescents conceived via IVF or ICSI may exhibit higher rates of altered body composition compared to naturally conceived peers. IVF-conceived offspring show a tendency toward increased adiposity, with higher central or peripheral fat mass despite similar body mass index (BMI) or stature [105]. This “catch-up” growth pattern in infancy and early childhood has been linked to an adverse cardiometabolic profile later in life [106].

Blood pressure alterations have also been reported. In some cohorts, IVF-conceived children were found to be twice as likely to present with high systolic or diastolic blood pressure compared to controls, even after adjustment for parental subfertility [107]. Interestingly, vascular dysfunction has been observed in adolescents conceived via IVF/ICSI but not in those born from subfertile couples who did not require ART procedures, suggesting that embryo manipulation could be associated with these changes [108].

Anomalies in lipid metabolism constitute another concern. Higher maternal estradiol levels during ovarian stimulation have been linked to dyslipidemia in newborns, including increased total and LDL cholesterol and reduced HDL levels [109]. While a few studies have described more favorable outcomes, the overall picture suggests that ART-conceived infants show grater variability in their metabolic profiles [110].

Finally, glucose metabolism may be affected. IVF-conceived children can have slightly higher fasting glucose levels compared to naturally conceived controls [107]. In summary, ART-conceived children may be predisposed to insulin resistance, altered lipid profile, hypertension, and obesity.

### 5.2. Neurodevelopmental Outcomes

The developing brain is particularly vulnerable to environmental and epigenetic perturbations. Studies on neurodevelopmental anomalies in ART-conceived children remain debated. Some prospective studies using validated developmental scales have shown no differences in cognitive, motor, or language abilities compared to controls [111], while population-based studies have highlighted the possibility of increased risks of specific disorders in the ART-conceived group.

Cerebral palsy (CP) was associated with IVF in a large population-based cohort study, though much of the risk was mediated by prematurity and multiple gestations [112]. When analyses were adjusted for preterm birth and multiple gestations, some studies reported that ART-conceived children (particularly those conceived by ICSI/IVF) had higher risk of mild neurodevelopmental delays [113]. A recent systematic review and meta-analysis showed that children born from ART are at higher risk of acquiring CP [114]. These findings suggest the importance of the availability of intensive care units at birth and long-term neurodevelopmental evaluation, especially in children born from ICSI.

Evidence is more consistent in relation to autism spectrum disorder (ASD). Several studies have reported a higher incidence of ASD in ICSI-conceived children compared to those conceived via conventional IVF or natural conception [106,115]. This suggests that both the bypassing of natural sperm selection and the manipulation of gametes may contribute to the risk. The role of paternal age and underlying infertility must also be considered, as both are independent risk factors for neurodevelopmental disorders [116].

### 5.3. Growth Trajectory

ART-conceived infants more frequently have low birth weight (LBW), particularly after fresh ETs [117]. LBW is a recognized risk factor for long-term metabolic disorders, including insulin resistance, hypertension, and obesity. Although frozen ET appears to reduce some of this risk, ART-conceived children often display accelerated postnatal “catch-up” growth, which may predispose them to central adiposity and adverse metabolic outcomes later in life [118].

## 6. Conclusions

In recent decades, ARTs have modified infertility treatment, providing hope to millions of couples worldwide. At the same time, their intersection with genomic imprinting has raised important questions about possible long-term health consequences. Previous studies reported an important association with IDs, particularly BWS, SRS, AS, and PPWS. These conditions are characterized by disruptions in parent-of-origin gene expression, most often linked to abnormal DNA methylation patterns or UPD. ART-related procedures, especially those involving the handling of gametes and embryos during critical windows of epigenetic reprogramming, may influence these molecular pathways. However, the risk of IDs is not attributable to a single intervention, but rather to the cumulative impact of multiple ART procedures applied during highly vulnerable developmental stages. This cumulative risk highlights the importance of optimizing each procedure to minimize epigenetic disruption.

Among the four major syndromes, BWS and SRS show the strongest and most consistent associations with ART. Several population-based studies have reported higher incidence rates in ART-conceived children, with hypomethylation at key ICRs being the most common molecular alteration. Conversely, evidence for AS and PWS is less consistent. Some studies suggest a possible link, particularly in cases involving ICSI and advanced maternal age, while others report no significant association. These discrepancies underscore the multifactorial nature of IDs, which probably result from an interplay between ART procedures, parental genetic susceptibility, and environmental exposures.

Overall, ARTs remain both safe and effective, but growing awareness of their potential epigenetic consequences is essential. Future research should prioritize longitudinal follow-up of ART-conceived cohorts and the development of national and international registries. Such efforts are essential to clarify the long-term health trajectories of these individuals and to clarify the effects of ART procedures in the presence of underlying parental infertility. For clinicians, counseling families about these emerging risks is particularly important when techniques with known epigenetic impacts are being considered. Ultimately, refining ART protocols to limit epigenetic disruption may help reduce the risk of IDs in future generations.

## Figures and Tables

**Table 1:** ART-related imprinting disorders: incidence, molecular mechanisms, symptoms, and prenatal findings.

Syndrome (Acronym)	Incidence (General Population)	Incidence (ART-Conceived)	Clinical Features	Prenatal Findings	Genetic/ Epigenetic Mechanisms	ART Association	References
Beckwith–Wiedemann Syndrome (BWS)	~1:10,340	4–15%; up to 10× increased risk	Macrosomia, macroglossia, omphalocele, visceromegaly, hypoglycemia, Wilms tumor risk	Fetal overgrowth, omphalocele, placentomegaly, mesenchymal dysplasia	ICR2 hypomethylation (50%), paternal UPD11 (20%), ICR1 GOM, CDKN1C mutations	Strong, especially with ICSI and cryopreservation	[30,31,35,36,37,38,39,40,41,42,43]
Silver–Russell Syndrome (SRS)	1:30,000–1:100,000	Up to 10× higher in some ART cohorts	IUGR, postnatal growth delay, triangular face, body asymmetry	IUGR, macrocephaly, facial dysmorphism	11p15.5 H19/IGF2 hypomethylation (30–60%), maternal UPD7 (5–10%)	Strong; especially linked to methylation errors	[15,50,56,57,58,59,60]
Angelman Syndrome (AS)	~1:15,000	~2× risk in ART-treated subfertile couples	Severe developmental delay, ataxia, speech impairment, happy demeanor	Not specific	UBE3A loss (maternal), deletions (65–75%), UPD, epimutations	Controversial; ART-related SNRPN hypomethylation	[28,44,45,46,47,48,49,50]
Prader–Willi Syndrome (PWS)	1:15,000–1:30,000	Mixed; up to 3.4× higher in some populations	Hypotonia, poor sucking, obesity, ID, hypogonadism, behavior issues	Reduced fetal movements, polyhydramnios	Paternal deletion (65–75%), maternal UPD (20–30%), ICR defects	Variable; possible link with maternal age, methylation errors	[30,50,51,52,53,54,55]
Temple Syndrome (TS14)	Very rare	Limited data	IUGR, neonatal hypotonia, truncal obesity, early puberty	Oligohydramnios, small placenta, IUGR	Maternal UPD14, LOM at MEG3/DLK1:IG-DMR	Plausible by a specific mechanism	[8,18,68]
Kagami–Ogata Syndrome (KOS14)	Very rare	Limited data	Polyhydramnios, respiratory failure, omphalocele, narrow thorax	Polyhydramnios, abdominal wall defects, narrow thorax	Paternal UPD14, GOM at MEG3/DLK1:IG-DMR, maternal deletions	Plausible by a specific mechanism	[8,18,69]
Transient Neonatal Diabetes Mellitus (TNDM)	Very rare	Sparse data	Hyperglycemia, low birth weight, macroglossia, abdominal wall defects	IUGR, macroglossia, abdominal wall defect	Paternal UPD6, LOM at PLAGL1-DMR	Insufficient data	[8,13,18]
Pseudohypoparathyroidism (PHP)	Rare	Limited data	PTH resistance, AHO features	Macrosomia (PHP1B), IUGR (PHP1A)	LOM at GNAS locus on chr20	Plausible epigenetic link	[8,18]

ART = assisted reproductive technologies; ID = imprinting disorder; BWS = Beckwith–Wiedemann syndrome; SRS = Silver–Russell syndrome; AS = Angelman syndrome; PWS = Prader–Willi syndrome; TS14 = Temple Syndrome; KOS14 = Kagami–Ogata syndrome; TNDM = transient neonatal diabetes mellitus; PHP = pseudohypoparathyroidism; UPD = uniparental disomy; ICR = imprinting control region; LOM = loss-of-methylation; GOM = gain-of-methylation; ICSI = intracytoplasmic sperm injection; IUGR = intrauterine growth restriction; AHO = Albright hereditary osteodystrophy; DMR = differentially methylated region.

## Data Availability

No new data were created or analyzed in this study.

## Abbreviations

ART	Assisted Reproductive Technology
AS	Angelman Syndrome
BPA	Bisphenol A
BWS	Beckwith–Wiedemann Syndrome
ESC	Embryonic Stem Cell
ET	Embryo Transfer
HDAC	Histone Deacetylase
ICR	Imprinting Control Region
ICSI	Intracytoplasmic Sperm Injection
ID	Imprinting Disorder
IVF	In Vitro Fertilization
MAGEL2	MAGE Family Member L2
MKRN3	Makorin Ring Finger Protein 3
NAD	Nicotinamide Adenine Dinucleotide
NECDIN	Neuronal Differentiation Protein
PGT	Preimplantation Genetic Testing
PWS	Prader–Willi Syndrome
SAM	S-Adenosylmethionine
SNRPN	Small Nuclear Ribonucleoprotein Polypeptide N
SNV	Single-Nucleotide Variant
SRS	Silver–Russell Syndrome
UBE3A	Ubiquitin Protein Ligase E3A
UPD	Uniparental Disomy
snRNA	Small Nucleolar RNA
